# SGLT2 inhibitor dapagliflozin treats heart failure with preserved ejection fraction via the SIRT1/PGC-1α pathway: Dapagliflozin is associated with improvements in HFpEF phenotypes

**DOI:** 10.3724/abbs.2026078

**Published:** 2026-05-28

**Authors:** Shiwen Zhang, Yansong Cui, Jingwen Chen, Shuaishuai Zhou, Yujiao Zhang, Kuan Li, Yinglong Hou

**Affiliations:** 1 Department of Cardiology Shandong Provincial Qianfoshan Hospital Shandong University Jinan 250014 China; 2 Department of General Medicine The Affiliated Hospital of Xuzhou Medical University Xuzhou 221002 China; 3 National Key Laboratory for Innovation and Transformation of Luobing Theory; The Key Laboratory of Cardiovascular Remodeling and Function Research Chinese Ministry of Education Chinese National Health Commission and Chinese Academy of Medical Sciences; Department of Cardiology Qilu Hospital of Shandong University Jinan 250014 China; 4 Department of Cardiology The Affiliated Hospital of Xuzhou Medical University Xuzhou 221002 China; 5Department of Cardiology Fengxian People’s Hospital Xuzhou 221700 China; 6 Clinical Medical Institute Xinjiang Medical University Urumqi 830000 China

**Keywords:** HFpEF, dapagliflozin, SGLT2 inhibitor, SIRT1/PGC-1α/Mfn-2 pathway, mitochondrial biosynthesis

## Abstract

Sodium-glucose cotransporter 2 inhibitors (SGLT2i) have demonstrated clinical benefits in heart failure with preserved ejection fraction (HFpEF), yet the underlying mechanisms remain poorly defined. Given that mitochondrial dysfunction represents a central feature of HFpEF pathophysiology, we investigate whether modulation of mitochondrial homeostasis contributes to the cardioprotective effects of dapagliflozin. Using a Dahl salt-sensitive rat model of HFpEF, we find that dapagliflozin markedly improves diastolic function and attenuates cardiac hypertrophy, fibrosis, and apoptosis. These beneficial effects are accompanied by significant restoration of mitochondrial structure and function. Consistently, in an *in vitro* HFpE model, dapagliflozin enhances mitochondrial respiratory capacity in cardiomyocytes, indicating a direct mitochondrial regulatory effect. Mechanistically, integrative transcriptomic and experimental analyses identify the SIRT1/PGC-1α/Mitofusin-2 (Mfn-2) signaling axis as a critical pathway suppressed in HFpEF but reactivated following dapagliflozin treatment. Activation of this pathway promotes mitochondrial biogenesis and improves mitochondrial dynamics, thereby preserving cardiomyocyte homeostasis. Collectively, our findings reveal that dapagliflozin exerts cardioprotective effects in HFpEF by restoring mitochondrial homeostasis through the SIRT1/PGC-1α/Mfn-2 axis, providing mechanistic insight into SGLT2i-mediated benefits and highlighting mitochondrial regulation as a potential therapeutic strategy for HFpEF.

## Introduction

Heart failure (HF), characterized by its complexity and heterogeneity, is a major global health concern [1]. Heart failure with preserved ejection fraction (HFpEF, EF ≥ 50%) accounts for approximately half of all HF cases, significantly impacting quality of life and increasing healthcare burdens [2]. With the aging global population, HFpEF is expected to become the most prevalent form of HF [3]. Despite its increasing incidence, effective treatment options remain limited. However, recent clinical trials on sodium-glucose cotransporter 2 inhibitors (SGLT2is) have provided promising therapeutic prospects [ 4– 7] .

Dapagliflozin, an SGLT2 inhibitor, is a novel antidiabetic drug that has demonstrated unexpected benefits for HFpEF patients [8]. The DELIVER trial reported an 18% reduction in cardiovascular death or worsening heart failure in HFpEF patients, along with a significant improvement in the quality of life and extended event-free survival [8]. The PRESERVED-HF trial showed that dapagliflozin rapidly improved symptoms and physical activity in HFpEF patients within 12 weeks, although its precise molecular mechanism remains unclear [9]. The risk factors of HFpEF include diabetes, obesity, hypertension, and aging [10], with mitochondrial dysfunction being a common pathological feature. Therefore, investigating the role of mitochondria in HFpEF, particularly in mitochondrial biogenesis pathways, represents a promising research direction [11].

Sirtuin 1 (SIRT1) is a crucial regulator of mitochondrial biogenesis and antioxidant defense. It deacetylates various non-histone targets, including peroxisome proliferator-activated receptor gamma coactivator 1-alpha (PGC-1α) [12]. PGC-1α activates transcription factors such as nuclear factor erythroid 2-related factor 2 (NRF-2), which binds to mitochondria-related gene promoters and regulates electron transport chain complex expression [13]. Our previous study identified SIRT1 and PGC-1α as elevated biomarkers in HFpEF patients’ serum, suggesting their diagnostic potential, but their roles in HFpEF pathogenesis remain unexplored [14].

In this study, HFpEF was induced in Dahl salt-sensitive rats via a high-salt diet. The effects of dapagliflozin on cardiac function, myocardial injury, mitochondrial biogenesis, and cardiac energy metabolism were evaluated. Additionally, dapagliflozin was found to enhance mitochondrial respiration in an HFpEF cell model using high glucose plus palmitic acid (HG + PA)-induced adult mouse ventricular myocytes (AMVMs). Finally, we demonstrated that dapagliflozin exerts its therapeutic effects through a SIRT1/PGC-1α/ Mitofusin-2 (Mfn-2) signaling axis, providing a potential avenue for developing novel HFpEF treatments.

## Materials and Methods

### Experimental animals

This study was approved by the Ethics Committee of the First Affiliated Hospital of Shandong First Medical University (approval No. 2019S026). All animal procedures complied with the Institutional Animal Care and Use Committee guidelines. Clinical trial registration was not applicable. Seven-week-old male Dahl salt-sensitive rats (Charles River, Beijing, China) were fed either a 0.3% NaCl (low-salt, D10001i, Research Diets) or 8% NaCl (high-salt, D05011703i, Research Diets) diet [ 15, 16] . After 7 weeks, high-salt diet animals were randomly assigned to receive either a placebo (water) or dapagliflozin via oral gavage. The dapagliflozin dosage (5 mg/kg/day) was determined based on a previous study [17]. Treatments were administered once daily for 2 weeks. EX-527 was administered via intraperitoneal injection at 5 mg/kg per day for 2 weeks [18]. After the final treatment, echocardiography was performed, followed by euthanasia and collection of heart and blood samples. The experimental protocol is illustrated in Supplementary Figure S1. Consistent with a previous study [17], we used 5 mg/kg/day in rats. Using body‑surface‑area scaling, 5 mg/kg in rats approximates ~0.8 mg/kg in humans (~57 mg/day for a 70‑kg adult), which exceeds the approved 10 mg/day due to interspecies pharmacokinetics differences; rodents typically require higher mg/kg to match exposure.

### Echocardiography and Doppler imaging

Rats were anesthetized with isoflurane and subjected to transthoracic echocardiography using a 13-MHz transducer. Cardiac images were acquired in the parasternal long-axis view to assess systolic function and in the apical four-chamber view to evaluate diastolic function. Heart rate was maintained between 425 and 475 beats per minute during image acquisition. Transmitral inflow velocities (E and A waves) were measured at the level of the mitral valve leaflets using pulsed-wave Doppler. Tissue Doppler imaging was performed at the septal mitral annulus to obtain the early diastolic velocity (e′). Left ventricular posterior wall thickness at diastole (LVPWd) was measured in M-mode using Vevo software. All measurements were obtained from at least three consecutive cardiac cycles and averaged for analysis. Data analysis was performed in a blinded manner to ensure objectivity.

### Tail-cuff blood pressure recordings

Non-invasive blood pressure measurements were performed on conscious rats using a CODA instrument (Kent Scientific, Torrington, USA). Prior to testing, rats were acclimated to short-term restraint. Blood pressure was recorded over at least three consecutive days, with each reading averaged from a minimum of five measurements.

### Exercise tolerance test

Rats were subjected to a treadmill running test to evaluate exercise capacity. Briefly, rats were placed on a motorized treadmill and run at a constant speed of 20 m/min. Electrical stimulation was applied at the rear of the treadmill (0.5 mA, 1 Hz, 200 ms pulse duration) to encourage running. Running time and total distance were recorded. Exhaustion was defined as the inability of the rat to resume running and its remaining on the shock grid for more than 10 s despite repeated electrical stimulation (at least three consecutive stimuli).

### Organ mass measurement

Rats were weighed and deeply anesthetized with sodium pentobarbital (50 mg/kg, intraperitoneally) prior to euthanasia. The chest cavity was opened, and the heart was rapidly excised and perfused with ice-cold phosphate-buffered saline (PBS) via the left ventricle to remove residual blood. Excess surface moisture was gently removed using filter paper. The atria were discarded, and the ventricles were isolated and weighed. Lung tissues were collected, briefly rinsed in PBS, and blotted dry to obtain wet weight. Samples were then dried at 37°C for 24 h to constant weight, and dry weight was recorded.

### TUNEL staining

TUNEL staining was performed to detect apoptotic cells in rat heart tissues. Briefly, rat hearts were harvested, fixed in 4% paraformaldehyde overnight at 4°C, embedded in paraffin, and sectioned at 4–5 μm thickness. Paraffin sections were deparaffinized in xylene and rehydrated through a graded ethanol series. Antigen retrieval was performed by heating sections in citrate buffer (pH 6.0) at 95–100°C for 10–15 min, followed by cooling to room temperature. Sections were then permeabilized with proteinase K (10 μg/mL, HY-108717; MedChemExpress, Monmouth Junction, USA) for 15–30 min at room temperature. After washing with PBS, sections were incubated with TUNEL reaction mixture (HY-K1078; MedChemExpress) according to the manufacturer’s instructions in a humidified chamber at 37°C for 60 min in the dark. Negative controls were prepared by omitting terminal deoxynucleotidyl transferase, while positive controls were treated with DNase I to induce DNA strand breaks prior to labeling. Following incubation, sections were rinsed with PBS and counterstained with DAPI (0.2 μg/mL, HY-D0814; MedChemExpress) to visualize nuclei.

TUNEL-positive cells were quantified in at least 5 randomly selected fields per section using ImageJ software (version 2.0; NIH, Bethesda, USA), and the percentage of apoptotic cells was calculated as the ratio of TUNEL-positive nuclei to total nuclei.

### Histological analysis

Rat hearts were excised, fixed in 4% paraformaldehyde in PBS overnight at 4°C, and embedded in paraffin. Serial sections (5 μm) were prepared for histological staining. Hematoxylin-eosin (HE) staining was performed to evaluate cardiomyocyte morphology and hypertrophy, and Masson’s trichrome staining was used to assess myocardial fibrosis, according to the manufacturers’ protocols (G1005 and G1006; Servicebio, Wuhan, China). Wheat germ agglutinin (WGA) staining was conducted to determine cardiomyocyte cross-sectional area (G1249; Servicebio). All stained sections were scanned using an Olympus VS200 slide scanning system (Olympus, Tokyo, Japan). Quantitative analysis was performed using ImageJ software, with five randomly selected microscopic fields per heart analyzed in a blinded manner.

### Transmission electron microscopy

Rat heart samples were fixed overnight at 4°C in a solution of 2% glutaraldehyde and 1% tannic acid in 0.1 M sodium cacodylate (pH 7.3). After three rinses in sodium cacodylate buffer, the samples were incubated in 2% osmium tetroxide for 2 h at room temperature. Following another 3 rinses, samples were treated with 1% uranyl acetate for 15 min, rinsed twice in distilled water, and embedded in 3% agarose at 45°C. Agarose blocks were dehydrated in a graded acetone series and embedded in Spurr Low Viscosity Medium. After polymerization overnight at 65°C, ultrathin sections (70–90 nm) sections were cut and visualized using a transmission electron microscope (Philips CM-10; Philips, Amsterdam, Netherlands). Micrographs were recorded on Kodak 4489 film. Mitochondrial density, area, and circumference were quantified using ImageJ 2.0 across 5 microscopic fields per heart, with results expressed as mitochondria per 100 μm ^2^.

### Enzyme-linked immunosorbent assays

Serum samples from each group of rats were collected to measure N-terminal pro-brain natriuretic peptide (NT-proBNP) levels. NT-proBNP concentrations were determined using a commercial ELISA kit (H334-1; Nanjing Jiancheng Bioengineering Institute, Nanjing, China) according to the manufacturer’s instructions. Briefly, standards and serum samples were added to the microplate wells and incubated as instructed, followed by the addition of enzyme conjugate and substrate solution. The reaction was terminated with stop solution, and absorbance was measured at 450 nm using a microplate reader. NT-proBNP concentrations were calculated based on a standard curve generated from known standards.

### RNA-seq and data analysis

Total RNA was extracted from cardiac tissues of control and HFpEF rats using TRIzol reagent (10606ES60; Yeasen, Shanghai, China). RNA quality and integrity were evaluated using agarose gel electrophoresis and an Agilent Bioanalyzer, and samples with RNA integrity number (RIN) ≥ 7.0 were used for library construction. Three biological replicates per group were included. Sequencing libraries were prepared and subjected to paired-end sequencing on an Illumina platform by LC-Bio (Hangzhou, China). Raw reads were filtered to remove adapters and low-quality sequences, and clean reads were aligned to the reference genome using HISAT2. Gene expression levels were quantified as FPKM values. Differential expression analysis was performed using DESeq2, with thresholds set at |fold change| > 1.5 and adjusted *P*-value (false discovery rate, FDR) < 0.05. GO and KEGG pathway enrichment analyses were conducted to identify significantly enriched biological functions and pathways using the LC-Bio Cloud Platform.

### Real-time quantitative polymerase chain reaction

Total RNA was extracted from rat heart tissues using TRIzol reagent according to the manufacturer’s instructions. RNA concentration and purity were determined spectrophotometrically. For reverse transcription, 1 μg of total RNA was converted into cDNA using the Hifair II 1st Strand cDNA Synthesis SuperMix for qPCR (11123ES60; Yeasen). RT-qPCR was performed using a SYBR Green system (Q311-03; Vazyme, Nanjing, China) on a real-time PCR instrument. Each 10 μL reaction mixture contained 5 μL of 2× SYBR Green PCR Master Mix, 0.2 μL of forward primer (10 μM), 0.2 μL of reverse primer (10 μM), 1 μL of cDNA template, and 3.6 μL of nuclease-free water. The final primer concentration was 0.2 μM. The primers are listed in Supplementary Table S1.

The PCR amplification conditions were as follows: initial denaturation at 95°C for 30 s, followed by 40 cycles of denaturation at 95°C for 10 s and annealing/extension at 60°C for 30 s. A melting curve analysis was performed to confirm the specificity of amplification. Gene expression levels were normalized to *β-actin* and calculated using the 2 ^−ΔΔCt^ method. All reactions were performed in triplicate.

### Western blot analysis

Total protein was extracted from rat heart tissue using RIPA lysis buffer (PC103; Yamei, Shanghai, China) and a protease inhibitor cocktail (GRF101; Yamei). The tissue was homogenized and centrifuged at 14,000 *g* for 15 min at 4°C. The supernatant was collected, and the protein concentration was determined using a BCA assay kit (ZJ101; Yamei). Proteins were separated by SDS-PAGE and transferred to polyvinylidene difluoride (PVDF) membranes (IPVH00010; Millipore, Billerica, USA). Membranes were blocked with 5% BSA for 2 h and incubated overnight at 4°C with primary antibodies against PGC-1α (1:1000, 2178S; CST, Danvers, USA), SIRT1 (1:1000, 8469S; CST), Mitofusion-2 (1:1000, 9482S; CST), and β-actin (1:5000, 4967S; CST). After washing with TBST (PS103; Yamei) for three times, the membranes were incubated for 1 h with a secondary antibody, IgG goat anti-rabbit (1:10,000, SA00001-2; Proteintech, Wuhan, China). Blots were visualized using a gel imaging system from Shanghai Tannon Scientific Instruments Co., Ltd. (Shanghai, China).

### Isolation and culture of adult mouse cardiomyocytes

Adult mouse cardiomyocytes were isolated using a standard Langendorff perfusion method. Briefly, mice were deeply anesthetized with sodium pentobarbital (30 mg/kg, intraperitoneally) prior to euthanasia. Hearts were rapidly excised and cannulated via the aorta for retrograde perfusion.

The hearts were first perfused with Ca ^2+^-free Krebs-Henseleit (KH) buffer (118 mM NaCl, 4.7 mM KCl, 1.2 mM MgSO _4_, 1.2 mM KH _2_PO _4_, 25 mM NaHCO _3_, 10 mM HEPES, and 11.1 mM glucose, pH 7.4) at 37°C for 5 min to remove blood. Subsequently, enzymatic digestion was performed using KH buffer containing collagenase type II (0.5 mg/mL; Worthington, Lakewood, USA) and 0.1 mM CaCl _2_ for 15–20 min until the tissue became softened. The left ventricles were then excised, minced into small pieces, and gently triturated to dissociate cardiomyocytes. The cell suspension was filtered through a 100-μm nylon mesh and subjected to gradual Ca ^2+^ reintroduction to a final concentration of 1.0 mM. Isolated cardiomyocytes were resuspended in plating medium [M199 (11150067; Gibco, Waltham, USA) supplemented with 5% fetal bovine serum (A5256701; Gibco), 1% penicillin/streptomycin (15140122; Gibco), and 10 mM 2,3-butanedione monoxime (B0753; Merck, Darmstadt, Germany)] and seeded into laminin-coated culture dishes. After attachment, cells were maintained in serum-free medium. Cell viability was assessed by morphology, and only rod-shaped cardiomyocytes with clear striations were considered viable. A successful isolation was defined as ≥80% rod-shaped cells.

### “Double Damage” HFpEF cell model

In the “double-hit” model, cells were treated with high glucose (12.5 mM, G8270; Merck) and palmitic acid (0.2 mM, P0500; Merck) for 12 h. Depending on the experimental group, dapagliflozin (10 μM; AstraZeneca, Cambridge, UK), SR-18292 (a PGC-1α inhibitor, 25 μM; Macklin, Shanghai, China), or EX-527 (10 μM; MedChemExpress) was administered in combination with high glucose and palmitic acid treatment. Control cells were maintained in serum-free medium containing less than 0.1% DMSO, which had no detectable effect on cell viability [19].

### NADP(H) and ATP assays

NADP(H) and ATP levels in myocardium and AMVMs were measured using the NADP(H) Assay Kit (S0179; Beyotime, Nantong, China) and ATP Assay Kit (S0026; Beyotime), respectively, following the manufacturer’s instructions.

### Seahorse assay

For mitochondrial respiration measurements, 500–800 AMVMs per well or 70%–80% confluent neonatal mouse ventricular myocytes (NMVMs) were seeded in laminin-coated Seahorse XFe96 microplates (103798-100; Agilent, Santa Clara, USA) and cultured for 30 min or 4 days. Before the experiment, the medium was replaced with XFe base medium (Agilent) containing glucose, pyruvate, and glutamine and then incubated at 37°C without CO _2_ for 30 min to equilibrate the temperature and pH. The initial oxygen consumption rate (OCR) was measured, followed by sequential injections of oligomycin (1 μM), carbonyl cyanide 4-(trifluoromethoxy)phenylhydrazone (FCCP, 1 μM), and rotenone/antimycin A (0.5 μM each) to evaluate ATP-linked OCR, maximal respiration, and non-mitochondrial respiration. For the extracellular acidification rate (ECAR), glucose (10 mM), oligomycin (1 μM), and 2-DG (50 mM) were added sequentially. Analysis was conducted with at least three technical replicates [20].

### Statistical analysis

All data are presented as the mean ± SEM. Statistical analyses were performed using one-way ANOVA with Bonferroni’s *post hoc* test. Statistical significance was set at *P*  < 0.05. All analyses were conducted using GraphPad Prism 8.0 software (GraphPad Software, La Jolla, USA).

## Results

### Dapagliflozin improves cardiac diastolic function and alleviates functional impairment in HFpEF rats

**Figure 1:**
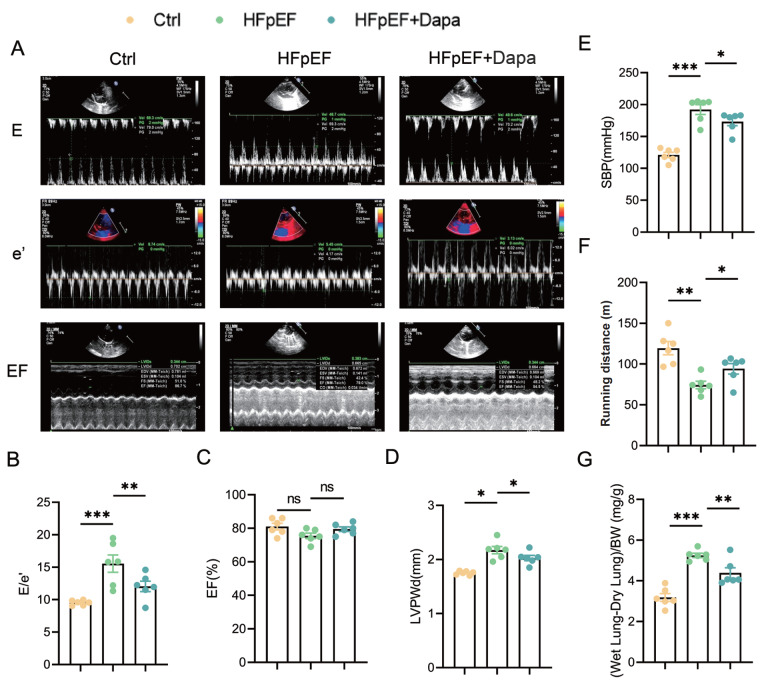
Figure 1 Dapagliflozin improves cardiac diastolic function and alleviates HFpEF symptoms in HFpEF rats (A) Representative pictures of echocardiography. (B‒D) Statistical analysis of EF (B), E/e’ (C) and LVPWd (D) (n = 6). (E) Statistical analysis of non-invasive tail artery blood pressure (n = 6). (F) Statistical analysis of running distance (n = 6). (G) Statistical analysis of wet lung-dry lung/ body weight (n = 6). EF: ejection fraction, LVPWd: left ventricular posterior wall diastolic. SBP: Systolic Blood Pressure. *P < 0.05, **P < 0.01, ***P < 0.001. ns, not significant.

We first established an HFpEF rat model using established protocols, which was validated by echocardiography. Compared with controls, HFpEF rats exhibited significantly elevated E/e′ values, preserved ejection fraction (EF), and increased left ventricular posterior wall thickness at diastole (LVPWd) and systolic blood pressure (SBP) ( Figure 1A–E), confirming successful model establishment. Given that HFpEF is characterized by exercise intolerance and pulmonary congestion secondary to diastolic dysfunction, we next evaluated these functional parameters. HFpEF rats displayed markedly reduced exercise capacity and a significantly increased lung wet-to-dry weight ratio normalized to body weight, indicative of pulmonary congestion ( Figure 1F,G). Following two weeks of treatment with dapagliflozin (5 mg/kg), HFpEF rats showed significant reductions in E/e′, LVPWd, and diastolic blood pressure compared with untreated HFpEF rats, whereas EF remained unchanged ( Figure 1A–E). In addition, exercise capacity was improved and pulmonary congestion was alleviated ( Figure 1F,G). These findings indicate that dapagliflozin effectively ameliorates diastolic dysfunction and associated functional impairment in HFpEF.

### Dapagliflozin attenuates myocardial injury and remodeling in HFpEF rats

**Figure 2:**
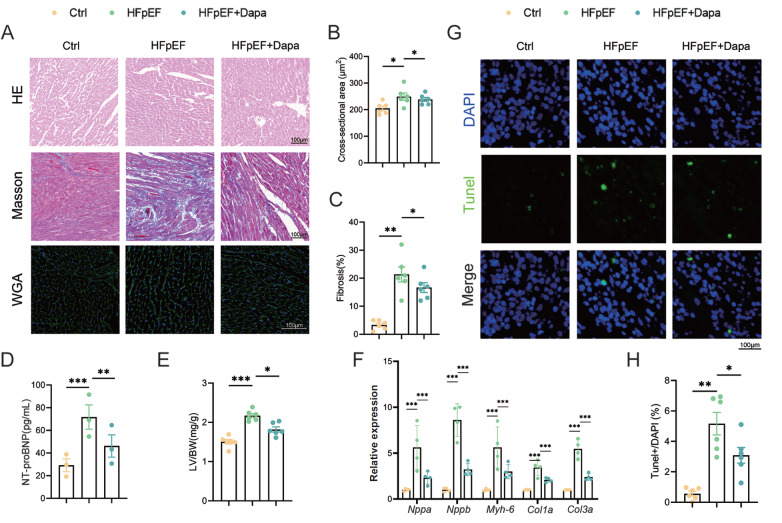
Figure 2 Dapagliflozin attenuates myocardial injury in HFpEF rats (A) Representative pictures of HE and Masson staining to observe myocardial hypertrophy and fibrosis in rats of each group. The scale bar is 100 μm. (B,C) Statistical analysis of cross-sectional area (E) and fibrosis (F) (n = 6). (D) Statistical analysis of serum NT-proBNP (n = 6). (E) Statistical analysis of the mass ratio of HW/BW (heart weight to body weight) (n = 6). (F) mRNA levels of Nppa, Nppb, Myh-6, Col1a, and Col3a (n = 4). (G) Representative pictures of TUNEL staining. The scale bar is 100 μm. (H) Statistical analysis of Tunel+/DAPI (n = 6). NT-proBNP: NT-pro brain natriuretic peptide. *P < 0.05, **P < 0.01, ***P < 0.001.

Histological analysis revealed pronounced myocardial injury in HFpEF rats, characterized by cardiomyocyte disorganization, hypertrophy, and increased interstitial fibrosis. In contrast, dapagliflozin treatment markedly improved myocardial architecture, as evidenced by reduced fiber disarray and attenuation of fibrosis ( Figure 2A–C). Consistently, HFpEF rats exhibited significantly elevated serum NT-proBNP levels and increased heart weight-to-body weight ratios, both of which were significantly reduced following dapagliflozin treatment ( Figure 2D,E), indicating attenuation of cardiac hypertrophy. At the molecular level, qPCR analysis demonstrated upregulation of hypertrophy-related genes ( *Nppa*, *Nppb* and *Myh6*) and fibrosis-associated genes ( *Col1a* and *Col3a*) in HFpEF rats, which were significantly suppressed by dapagliflozin ( Figure 2F). Furthermore, TUNEL staining revealed a marked increase in cardiomyocyte apoptosis in HFpEF rats, whereas dapagliflozin treatment significantly reduced apoptotic cell numbers ( Figure 2G,H). Together, these results indicate that dapagliflozin alleviates myocardial injury, remodeling, and apoptosis in HFpEF.

### Dapagliflozin activates the SIRT1/PGC-1α signaling pathway and improves mitochondrial function in HFpEF rats

**Figure 3:**
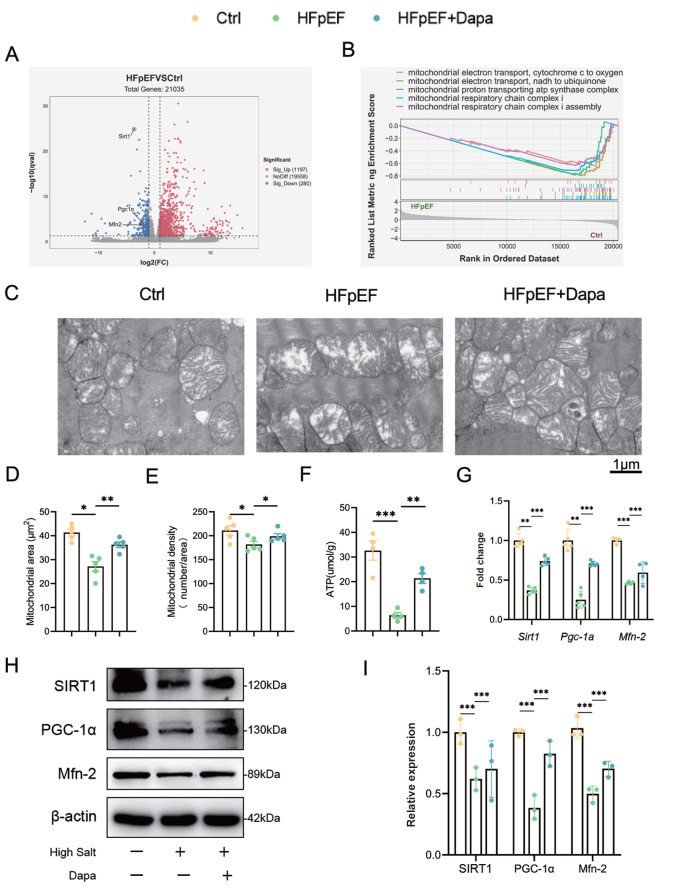
Figure 3 Dapagliflozin activates mitochondrial regeneration and the SIRT1/PGC-1α signaling pathway in HFpEF rats (A) Volcano plot of DEGs. (B) Gene Set Enrichment Analysis of DEGs. (C) Representative micrographs of heart tissue sections examined by transmission electron micrographs. The scale bar is 1 μm. (D) Statistical analysis of mitochondrial area (n = 6). (E) Statistical analysis of mitochondria number (n = 6). (F) Statistical analysis of ATP content in mitochondria of myocardial tissue (n = 6). (G) mRNA levels of Sirt1, Pgc-1a, and Mfn-2 in the heart (n = 4). (H,I) Representative western blots and statistical analysis of SIRT1, PGC-1α and Mfn-2 in the heart (n = 3). *P < 0.05, **P < 0.01, ***P < 0.001.

To further elucidate the molecular mechanisms underlying HFpEF, RNA sequencing was performed on cardiac tissues. A total of 1972 upregulated and 260 downregulated genes were identified in HFpEF rats compared with controls ( Figure 3A and Supplementary Figure S2A,B). Notably, genes involved in mitochondrial biogenesis and energy metabolism, particularly those within the SIRT1/PGC-1α/Mfn-2 signaling axis, were significantly downregulated. GO enrichment analysis revealed significant enrichment of pathways related to energy metabolism ( Supplementary Figure S2C), while gene set enrichment analysis (GSEA) demonstrated suppression of mitochondrial respiratory chain-related pathways ( Figure 3B). Consistently, KEGG pathway analysis identified multiple metabolism-associated pathways, including AMPK signaling, fatty acid metabolism, and TNF-α signaling ( Supplementary Table S2).

Transmission electron microscopy further demonstrated profound mitochondrial structural abnormalities in HFpEF rats. Compared with controls, cardiomyocyte mitochondria exhibited marked swelling, disrupted cristae, matrix rarefaction, and occasional vacuolation. Dapagliflozin treatment substantially restored mitochondrial ultrastructure, as evidenced by reduced swelling and improved cristae organization ( Figure 3C). Quantitative analysis confirmed significant reductions in mitochondrial density and surface area in HFpEF rats, both of which were partially restored following dapagliflozin treatment ( Figure 3D,E). Consistent with these structural changes, myocardial ATP levels were significantly decreased in HFpEF rats and markedly increased following dapagliflozin treatment ( Figure 3F), indicating improved mitochondrial energy metabolism. Given the central role of the SIRT1/PGC-1α/Mfn-2 pathway in regulating mitochondrial biogenesis and function, we further examined its expression. Both mRNA and protein levels of SIRT1, PGC-1α, and Mfn-2 were significantly downregulated in HFpEF rats but were robustly restored upon dapagliflozin treatment ( Figure 3G–I). Collectively, these results demonstrate that dapagliflozin alleviates mitochondrial structural and functional impairments in HFpEF, at least in part, through activation of the SIRT1/PGC-1α/Mfn-2 signaling pathway.

### Dapagliflozin enhances mitochondrial respiration in an *in vitro* HFpEF model via the SIRT1/PGC-1α pathway

**Figure 4:**
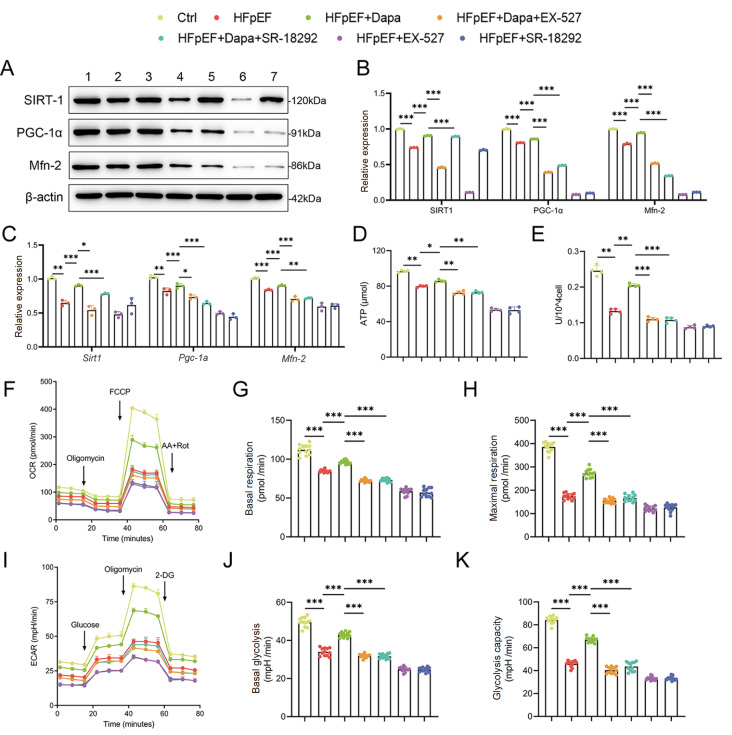
Figure 4 Dapagliflozin improves mitochondrial respiratory function through the SIRT1/PGC-1α pathway in the HFpEF cell model (A,B) Representative western blots and statistical analysis of SIRT1, PGC-1α and Mfn-2 in AMVMs (n = 3). (C) mRNA levels of Sirt1, Pgc-1a, and Mfn-2 (n = 3). (D) Statistical analysis of ATP content in mitochondria of AMVMs (n = 4). (E) Statistical analysis of NADP(H) content in mitochondria of AMVMs (n = 4). (F) Real-time mitochondrial respiration of AMVMs (n = 3 independent experiments). (G) Statistical analysis of basal OCR of AMVMs (n = 3 independent experiments). (H) Statistical analysis of maximal OCR of AMVMs (n = 3 independent experiments). (I) Real-time glycolytic capacity of AMVMs (n = 3 independent experiments). (J) Statistical analysis of basal ECAR of AMVMs (n = 3 independent experiments). (K) Statistical analysis of maximal ECAR of AMVMs (n = 3 independent experiments). *P < 0.05, **P < 0.01, ***P < 0.001.

To further validate the mechanistic findings, an *in vitro* HFpEF model was established using high glucose plus palmitic acid (HG + PA)-treated adult mouse cardiomyocytes. Consistent with *in vivo* observations, the expression of key components of the SIRT1/PGC-1α/Mfn-2 pathway was significantly suppressed in HFpEF cells, indicating impaired mitochondrial biogenesis ( Figure 4A–C). Treatment with Dapagliflozin markedly restored the expression of these genes. In contrast, pharmacological inhibition of SIRT1 and PGC-1α using EX-527 and SR-18292 further suppressed pathway activity and abolished the effects of dapagliflozin, confirming the involvement of this signaling axis ( Figure 4A–C). Functional assessment revealed that intracellular ATP and NADP(H) levels were significantly reduced in HFpEF cells, reflecting impaired mitochondrial respiration ( Figure 4D,E). Dapagliflozin treatment significantly increased both ATP and NADP(H) levels, whereas inhibition of SIRT1 or PGC-1α reversed these effects. To evaluate cellular bioenergetics, mitochondrial respiration and glycolytic function were assessed using Seahorse assay. HFpEF model cells exhibited marked reductions in basal respiration, ATP-linked respiration, maximal respiratory capacity, and spare respiratory capacity, as well as impaired glycolytic function. Dapagliflozin significantly improved both mitochondrial respiration and glycolytic capacity. However, these improvements were largely abolished upon inhibition of SIRT1 or PGC-1α ( Figure 4F–K). Taken together, these findings indicate that dapagliflozin enhances mitochondrial bioenergetics and metabolic flexibility in HFpEF cardiomyocytes through activation of the SIRT1/PGC-1α signaling pathway.

### Inhibition of SIRT1 attenuates the cardioprotective effects of dapagliflozin in HFpEF rats

**Figure 5:**
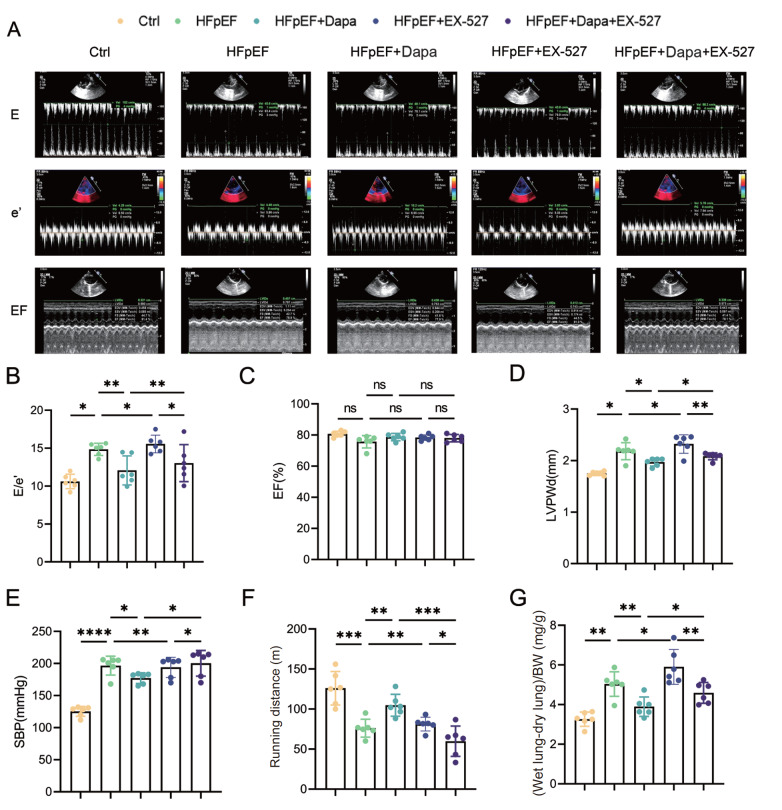
Figure 5 Inhibiting SIRT1 weakens the therapeutic effects of dapagliflozin on HFpEF rats (A) Representative pictures of echocardiography. (B‒D) Statistical analysis of EF (B), E/e’ (C) and LVPWd (D) (n = 6). (E) Statistical analysis of non-invasive tail artery blood pressure (n = 6). (F) Statistical analysis of running distance (n = 6). (G) Statistical analysis of wet lung-dry lung/ body weight (n = 6). *P < 0.05, **P < 0.01, ***P < 0.001. ns, not significant.

To determine whether the cardioprotective effects of dapagliflozin are mediated via SIRT1 signaling, HFpEF rats were treated with dapagliflozin in the presence or absence of EX-527. Echocardiographic analysis revealed that co-administration of EX-527 partially reversed the beneficial effects of dapagliflozin, as indicated by increased E/e′ and LVPWd values compared with dapagliflozin treatment alone ( Figure 5A,B). The EX-527 group exhibited the most severe diastolic dysfunction, whereas EF remained unchanged across groups ( Figure 5C). In addition, EX-527 markedly attenuated dapagliflozin-induced improvements in systolic blood pressure, exercise capacity, and pulmonary congestion ( Figure 5D–G). These findings suggest that SIRT1 activity is required for the cardioprotective effects of dapagliflozin in HFpEF.

### SIRT1 inhibition abolishes the beneficial effects of dapagliflozin on myocardial remodeling and apoptosis

**Figure 6:**
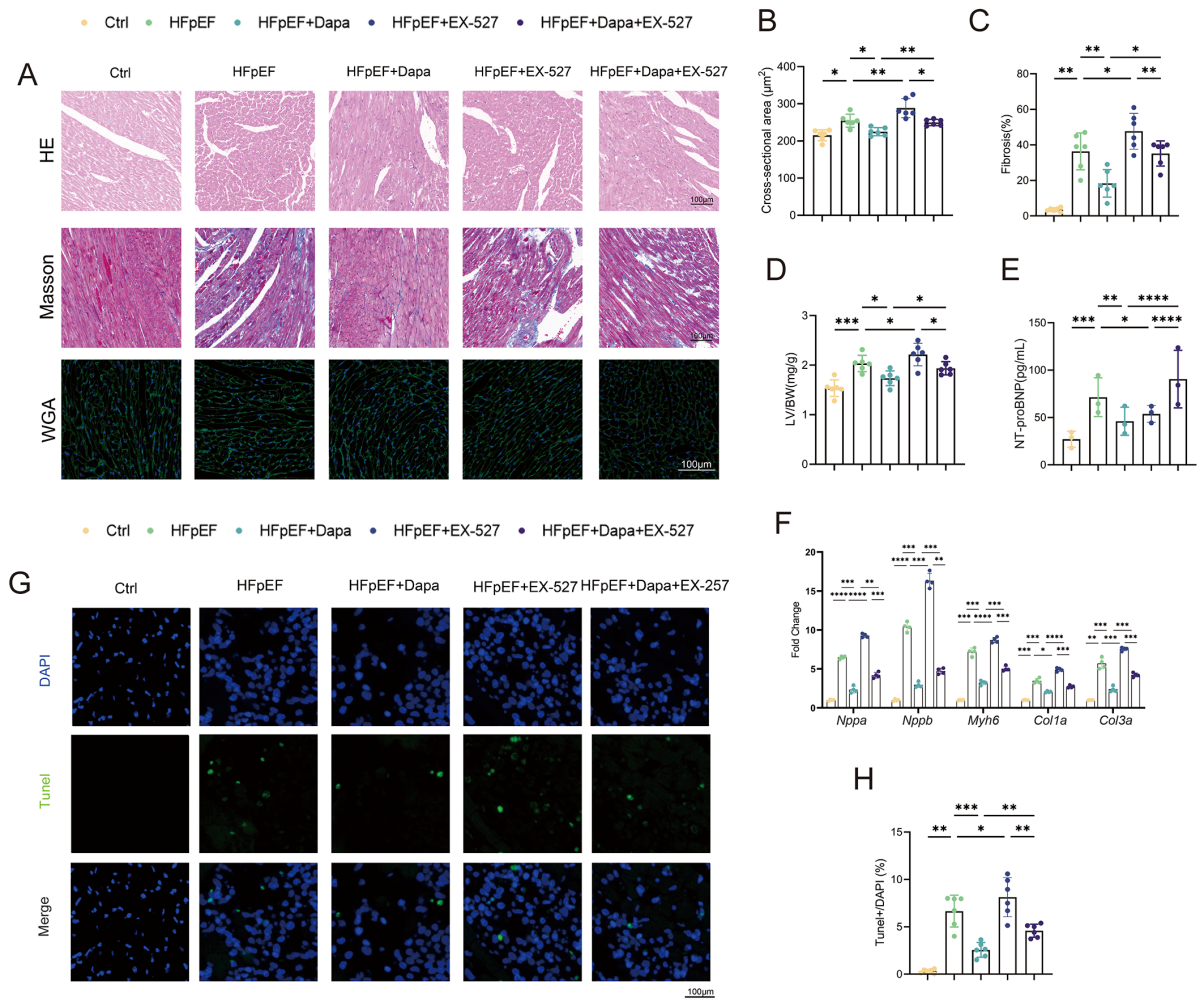
Figure 6 Inhibiting SIRT1 reverses the enhancement of myocardial hypertrophy and fibrosis by dapagliflozin (A) Representative pictures of HE and Masson staining to observe myocardial hypertrophy and fibrosis in rats of each group. The scale bar is 100 μm. (B,C) Statistical analysis of cross-sectional area (E) and fibrosis (F) (n = 6). (D) Statistical analysis of serum NT-pro brain natriuretic peptide (NT-proBNP) (n = 6). (E) Statistical analysis of the mass ratio of heart weight to body weight (n = 6). (F) mRNA levels of Nppa, Nppb, Myh-6, Col1a, and Col3a (n = 4). (G) Representative pictures of TUNEL staining. The scale bar is 100 μm. (H) Statistical analysis of TUNEL+/DAPI (n = 6). *P < 0.05, **P < 0.01, ***P < 0.001, ****P < 0.0001.

Histological analysis demonstrated that myocardial fiber disarray, cardiomyocyte hypertrophy, and interstitial fibrosis were significantly aggravated in the EX-527 + dapagliflozin group compared with the dapagliflozin group ( Figure 6A–C). The EX-527 group exhibited the most severe pathological alterations, further supporting a critical role of SIRT1 in mediating the protective effects of dapagliflozin. Similarly, serum NT-proBNP levels and heart weight-to-body weight ratios were significantly increased upon SIRT1 inhibition, indicating exacerbation of cardiac hypertrophy ( Figure 6D,E). Gene expression analysis of hypertrophy- and fibrosis-related markers was consistent with histological findings ( Figure 6F). Moreover, TUNEL staining revealed a significant increase in cardiomyocyte apoptosis in the EX-527 + dapagliflozin group compared with dapagliflozin alone ( Figure 6G,H). Collectively, these results demonstrate that inhibition of SIRT1 largely abolishes the beneficial effects of dapagliflozin on myocardial remodeling and apoptosis in HFpEF.

### Dapagliflozin treats HFpEF in rats via SIRT1/PGC-1α signaling pathway

**Figure 7:**
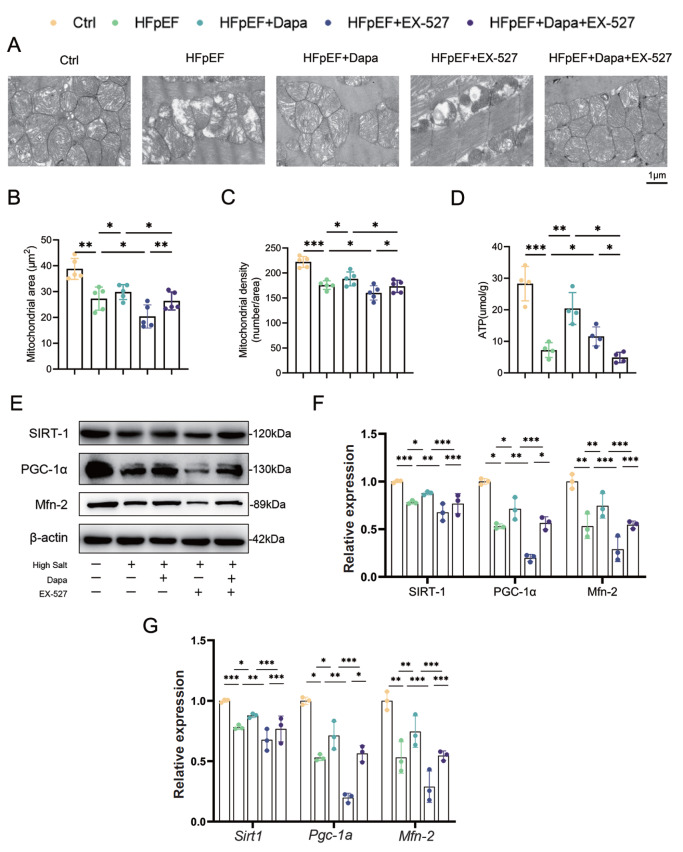
Figure 7 Dapagliflozin is associated with improvements in HFpEF phenotypes via the SIRT1/PGC-1α signaling pathway (A) Representative micrographs of heart tissue sections examined by transmission electron micrographs. The scale bar is 1 μm. (B) Statistical analysis of mitochondrial area (n = 6). (C) Statistical analysis of mitochondria number (n = 6). (D) Statistical analysis of ATP content in mitochondria of myocardial tissue (n = 6). (E) mRNA levels of Sirt1, Pgc-1a, and Mfn-2 in the heart (n = 6). (F,G) Representative western blots and statistical analysis of SIRT1, PGC-1α and Mfn-2 in the heart (n = 3). *P < 0.05, **P < 0.01, ***P < 0.001.

Transmission electron microscopy revealed that mitochondrial ultrastructure was markedly disrupted in the EX-527 + dapagliflozin group compared with the dapagliflozin group. Cardiomyocytes exhibited swollen mitochondria, disorganized cristae, matrix rarefaction, and increased vacuolation ( Figure 7A). The EX-527 group displayed the most severe mitochondrial damage, indicating that SIRT1 inhibition negates the protective effects of dapagliflozin on mitochondrial integrity ( Figure 7B,C). Consistently, myocardial ATP levels were significantly increased following dapagliflozin treatment but were markedly reduced upon co-administration of EX-527 ( Figure 7D), indicating impaired energy metabolism. Furthermore, both gene and protein expression analyses demonstrated that inhibition of SIRT1 significantly suppressed the SIRT1/PGC-1α/Mfn-2 signaling pathway, even in the presence of dapagliflozin ( Figure 7E–G). Together, these findings indicate that SIRT1 is essential for dapagliflozin-mediated improvements in mitochondrial structure and energy metabolism in HFpEF.

## Discussion

Mitochondrial dysfunction, characterized by impaired bioenergetics and increased oxidative stress, is a hallmark of cardiovascular disease [21]. In patients with heart failure with reduced ejection fraction (HFrEF) and diabetes mellitus, mitochondrial abnormalities have been well documented and are increasingly recognized as critical contributors to the pathophysiology of HFpEF [22]. Over the past two decades, the conceptual framework of HFpEF has evolved from a purely hemodynamic disorder to a complex systemic syndrome involving chronic inflammation [ 23, 24] , impaired angiogenesis [25], multiorgan dysfunction [26], and metabolic dysregulation [27]. At the cardiac level, HFpEF is associated with hypertrophy, fibrosis, excitation–contraction uncoupling, and defects in mitochondrial metabolism. These observations highlight mitochondrial biogenesis and metabolic regulation as promising therapeutic targets in HFpEF.

**Figure 8:**
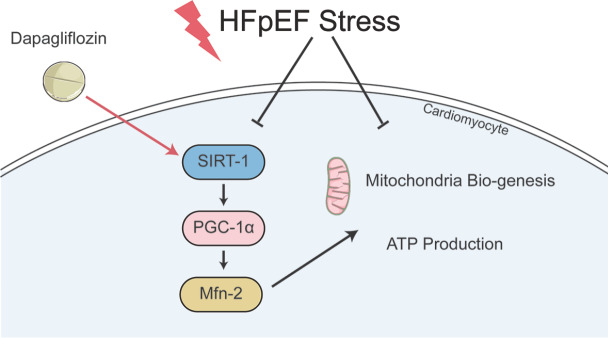
Figure 8 Diagram of the treatment of HFpEF with dapagliflozin via the SIRT1/PGC-1α pathway

In the present study, we demonstrate that the dapagliflozin exerts significant cardioprotective effects in HFpEF by improving diastolic function, attenuating myocardial remodeling, and restoring mitochondrial structure and function. Mechanistically, these effects are closely associated with activation of the SIRT1/PGC-1α/Mfn-2 signaling axis. Importantly, pharmacological inhibition of SIRT1 largely abolished the beneficial effects of dapagliflozin in both *in vivo* and *in vitro* models, supporting a central role for this pathway in mediating its therapeutic actions ( Figure 8).

Recent large-scale clinical trials, including EMPEROR-Preserved, DELIVER, and CANVAS, have established the clinical efficacy of SGLT2 inhibitors in HFpEF [ 8, 9] . However, the underlying mechanisms remain incompletely understood. Emerging evidence suggests that SGLT2 inhibitors exert pleiotropic effects on mitochondrial metabolism. For example, empagliflozin has been shown to protect against doxorubicin-induced cardiotoxicity through modulation of mitochondrial SIRT3 signaling and the formation of a TLR9-Beclin-1-SIRT3 complex, thereby regulating mitochondrial function and reactive oxygen species production [28]. In addition, SGLT2i has been reported to enhance mitochondrial respiratory chain activity, particularly complex II function, and to modulate inner mitochondrial membrane properties in ischemia–reperfusion models [ 29– 31] . Notably, increased expression of SIRT1 has also been observed following SGLT2 inhibitor treatment. Consistent with these findings, our results demonstrate that dapagliflozin restores SIRT1 expression and downstream mitochondrial regulatory pathways in HFpEF.

Beyond SIRT1, SGLT2 inhibitors have been shown to broadly regulate metabolic homeostasis. For instance, canagliflozin improves glucose-lipid metabolism, reduces adiposity, and enhances mitochondrial function by upregulating key regulators of mitochondrial biogenesis and fatty acid oxidation, including PGC-1α, NRF-2, and TFAM. These effects collectively promote mitochondrial efficiency and metabolic flexibility. Our findings extend these observations by demonstrating that SIRT1 inhibition reverses the therapeutic benefits of dapagliflozin in HFpEF, further supporting the notion that SIRT1-centered signaling networks play a pivotal role in SGLT2 inhibitor-mediated cardio-protection.

Consistent with previous reports, our ultrastructural analyses revealed marked mitochondrial damage in HFpEF, including swelling, cristae disruption, and matrix disorganization. These abnormalities were significantly ameliorated by dapagliflozin treatment, accompanied by restoration of ATP production and mitochondrial respiratory capacity. Similar mitochondrial protective effects have been reported in other tissues. For example, dapagliflozin improves synaptic plasticity and reduces mitochondrial oxidative stress in the hippocampus [ 32, 33] , while empagliflozin restores mitochondrial integrity in renal tubular cells under high-fat diet conditions [34]. Together, these findings support a conserved role for SGLT2 inhibitors in maintaining mitochondrial homeostasis across tissues.

In addition to mitochondrial pathways, our transcriptomic analysis identified multiple signaling cascades potentially contributing to the observed cardioprotective effects. The AMPK signaling pathway, a central regulator of cellular energy balance, was consistently enriched and is known to promote fatty acid oxidation and mitochondrial biogenesis. Furthermore, enrichment of pathways related to oxidative phosphorylation, the tricarboxylic acid cycle, and fatty acid metabolism suggests coordinated regulation of mitochondrial redox homeostasis, potentially involving SIRT3-mediated deacetylation mechanisms. In parallel, inflammatory pathways, including NF-κB, TNF, and cytokine-cytokine receptor interactions, were also significantly enriched, indicating that attenuation of inflammation may synergize with metabolic improvements [35]. These data suggest that SGLT2 inhibition exerts its therapeutic effects through integrated modulation of mitochondrial function, metabolic signaling, and inflammatory responses.

To further validate our findings, we employed a recently developed “double-damage” cellular model of HFpEF, induced by high glucose and palmitic acid. This model recapitulates key features of HFpEF cardiomyocytes, including preserved contractility and impaired diastolic function, without significant loss of cell viability. Using this model, we confirmed that mitochondrial respiration and oxidative phosphorylation were significantly impaired, whereas dapagliflozin treatment restored mitochondrial bioenergetics. These results provide additional mechanistic support for the role of mitochondrial dysfunction in HFpEF and its modulation by dapagliflozin.

Despite these findings, several limitations should be acknowledged. First, although our data strongly support an association between SIRT1 activation and the therapeutic effects of dapagliflozin, definitive causal relationships require further validation using genetic approaches, such as cardiomyocyte-specific *SIRT1* knockout or AAV-mediated gene silencing. Second, the Dahl salt-sensitive rat model and the HG + PA-induced cellular model do not fully recapitulate the complex systemic features of HFpEF, including aging, obesity, renal dysfunction, and vascular inflammation. Third, only male animals were included in this study, which may limit the generalizability of our findings, given the higher prevalence of HFpEF in females. Future studies incorporating sex differences and more clinically relevant models are warranted.

In conclusion, our study demonstrates that dapagliflozin ameliorates HFpEF by improving mitochondrial biogenesis and cardiac energy metabolism, at least in part through activation of the SIRT1/PGC-1α signaling pathway. These findings provide mechanistic insight into the cardioprotective effects of SGLT2 inhibitors and highlight mitochondrial regulation as a potential therapeutic target in HFpEF.

## Supporting information

741Supplementary_Material

## Supplementary Data

Supplementary data are available at *Acta Biochimica et Biophysica Sinica* online.
