# Supplementary material for: SGLT2 inhibitor dapagliflozin treats heart failure with preserved ejection fraction via the SIRT1/PGC-1α pathway: Dapagliflozin is associated with improvements in HFpEF phenotypes
Source: Acta Biochim Biophys Sin (Shanghai). 2026 May 28;58(7):1624–36. doi: 10.3724/abbs.2026078 (PMC13449090; doi:10.3724/abbs.2026078)
Supplement: Supplementary file 1 [file 741Supplementary_Material.docx]

**Supplementary Figure S1.** **Illustrations of animal experiments**

**Supplementary Figure S2. Results of RNA-seq of HFpEF rat heart tissue** (A) Statistics of upregulated and downregulated DEGs. (B) Heatmap of DEGs. (C) GO enrichment of DEGs.

**Supplementary Table S1. Primers used in this study**

| **Gene** | **Forward primer（5′-3′）** | **Reverse primer（5′-3′）** |
| --- | --- | --- |
| *GAPDH* | TGCCACTCAGAAGACTGTGG | TTCAGCTCTGGGATGACCTT |
| *PGC1α* | TATTCATTGTTCGATGTGTCGC | TGTCTGTAGTGGCTTGATTCAT |
| *SIRT1* | ATCTCCCAGATCCTCAAGCCA | CTTCCACTGCACAGGCACAT |
| *Mfn-2* | CACCGTCAAGAAGGATAAGCG | CAGTTCTGTATTCCTGTGGGTGTC |
| *ANP* | TTCCTCTTCCTGGCCTTTTG | TCTAGCAGGTTCTTGAAATCCATC |
| *BNP* | TCCAGGAGAGACTTCGAAATTC | GCAAGTTTGTGCTGGAAGATAA |
| *Col1a* | CGTGGAAACCTGATGTATGCTTG | CCTATGACTTCTGCGTCTGGTGA |
| *Col3a* | CGAGGTAACAGAGGTGAAAGAGG | TTTCACCTCCAACTCCAGCAAT |
| *β-Mhc* | CTGGTGGACAAGCTGCAGTTAA | CATCCTTAGGGTTGGGTAGCAC |

**Supplementary Table S2.** **KEGG enrichment pathway in RNA-seq**

| **NAME** | **SIZE** | **ES** | **NES** | **FDR.qval** |
| --- | --- | --- | --- | --- |
| propanoate metabolism | 30 | -0.7205319 | -2.6412153 | 0 |
| citrate cycle (tca cycle) | 28 | -0.69822216 | -2.5270712 | 0 |
| oxidative phosphorylation | 130 | -0.50657743 | -2.478381 | 0 |
| fatty acid degradation | 43 | -0.603085 | -2.4774616 | 0 |
| lipoic acid metabolism | 18 | -0.71531135 | -2.4149086 | 0.0004381 |
| valine, leucine and isoleucine degradation | 50 | -0.5452285 | -2.3570302 | 0.00036508 |
| 2 oxocarboxylic acid metabolism | 30 | -0.5532489 | -2.0995913 | 0.00058673 |
| butanoate metabolism | 24 | -0.5844598 | -2.0899036 | 0.00051339 |
| fatty acid biosynthesis | 18 | -0.6224029 | -2.028284 | 0.0011719 |
| carbon metabolism | 109 | -0.39143887 | -1.9513611 | 0.00364872 |
| pyruvate metabolism | 40 | -0.4777587 | -1.9346874 | 0.00331702 |
| peroxisome | 83 | -0.42183807 | -1.8963248 | 0.00445902 |
| thermogenesis | 221 | -0.32791802 | -1.8155681 | 0.00828635 |
| nicotine addiction | 32 | -0.47899064 | -1.7903845 | 0.0093107 |
| fatty acid metabolism | 60 | -0.41669884 | -1.7645619 | 0.01002443 |
| fatty acid elongation | 29 | -0.4615072 | -1.7565467 | 0.01011343 |
| tryptophan metabolism | 44 | -0.4315403 | -1.7495989 | 0.00981166 |
| diabetic cardiomyopathy | 199 | -0.33561936 | -1.6085186 | 0.02459638 |
| ppar signaling pathway | 75 | -0.3295679 | -1.5215132 | 0.03941044 |
| cardiac muscle contraction | 83 | -0.31732312 | -1.5060314 | 0.04284253 |
| linoleic acid metabolism | 27 | -0.39691654 | -1.4881436 | 0.04696167 |
| chemical carcinogenesis reactive oxygen species | 218 | -0.3119796 | -1.4581681 | 0.05673406 |
| glycolysis / gluconeogenesis | 57 | -0.32150835 | -1.416359 | 0.07212775 |
| beta alanine metabolism | 28 | -0.37615493 | -1.3468425 | 0.11026211 |
| retinol metabolism | 42 | -0.3223008 | -1.3226727 | 0.12099979 |
| retrograde endocannabinoid signaling | 139 | -0.27364582 | -1.3216745 | 0.11712942 |
| taste transduction | 55 | -0.30987155 | -1.3208733 | 0.11311944 |
| glyoxylate and dicarboxylate metabolism | 29 | -0.34913006 | -1.2694896 | 0.15575652 |
| fat digestion and absorption | 38 | -0.3086102 | -1.2460285 | 0.17482597 |
| non alcoholic fatty liver disease | 150 | -0.23757927 | -1.2181978 | 0.19981499 |
| metabolism of xenobiotics by cytochrome p450 | 45 | -0.2993271 | -1.1913351 | 0.22758928 |
| biosynthesis of unsaturated fatty acids | 32 | -0.29354855 | -1.15866 | 0.270101 |
| huntington disease | 287 | -0.21702448 | -1.1474087 | 0.28115562 |
| nitrogen metabolism | 16 | -0.35452172 | -1.1020122 | 0.35648292 |
| glycine, serine and threonine metabolism | 36 | -0.27172363 | -1.079926 | 0.3916984 |
| starch and sucrose metabolism | 24 | -0.29881528 | -1.0790554 | 0.38284782 |
| phenylalanine metabolism | 15 | -0.34648997 | -1.0556442 | 0.4215438 |
| pentose phosphate pathway | 29 | -0.2809535 | -1.0457969 | 0.43374008 |
| alpha linolenic acid metabolism | 23 | -0.29761818 | -1.0418286 | 0.4316611 |
| alanine, aspartate and glutamate metabolism | 35 | -0.2670142 | -1.0305605 | 0.4469557 |
| arachidonic acid metabolism | 59 | -0.24294333 | -1.0254508 | 0.4471921 |
| proximal tubule bicarbonate reclamation | 21 | -0.2877052 | -0.99199593 | 0.51734895 |
| spinocerebellar ataxia | 135 | -0.18927698 | -0.9470093 | 0.6119321 |
| olfactory transduction | 134 | -0.1855055 | -0.9168663 | 0.6701908 |
| cobalamin transport and metabolism | 17 | -0.28086746 | -0.8767744 | 0.74404526 |
| arginine biosynthesis | 21 | -0.2553207 | -0.8466014 | 0.78638065 |
| protein processing in endoplasmic reticulum | 162 | 0.61473966 | 2.0925536 | 0 |
| coronavirus disease covid 19 | 244 | 0.60520095 | 2.0829587 | 0 |
| n glycan biosynthesis | 48 | 0.68937767 | 2.0784647 | 0 |
| pertussis | 68 | 0.6387988 | 2.017344 | 0 |
| nf kappa b signaling pathway | 97 | 0.59862524 | 1.9550678 | 0.00055311 |
| various types of n glycan biosynthesis | 40 | 0.6602407 | 1.9507182 | 0.00046093 |
| osteoclast differentiation | 123 | 0.5828424 | 1.9359558 | 0.00039508 |
| yersinia infection | 134 | 0.5731347 | 1.9232587 | 0.00034569 |
| natural killer cell mediated cytotoxicity | 90 | 0.58997333 | 1.9119153 | 0.00030728 |
| african trypanosomiasis | 35 | 0.64958 | 1.8978132 | 0.00054988 |
| ribosome | 163 | 0.559062 | 1.8933419 | 0.00049989 |
| toll like receptor signaling pathway | 88 | 0.5812016 | 1.8825591 | 0.00061014 |
| leishmaniasis | 69 | 0.5909731 | 1.8725975 | 0.00077641 |
| staphylococcus aureus infection | 62 | 0.5963929 | 1.861578 | 0.00091771 |
| complement and coagulation cascades | 70 | 0.583991 | 1.860234 | 0.00091859 |
| rheumatoid arthritis | 80 | 0.58244145 | 1.8599951 | 0.00086118 |
| lysosome | 122 | 0.56135166 | 1.8564165 | 0.00086405 |
| phagosome | 155 | 0.54838675 | 1.8553022 | 0.00081605 |
| malaria | 48 | 0.61178213 | 1.845798 | 0.0009683 |
| lipid and atherosclerosis | 189 | 0.5317062 | 1.8289448 | 0.00105727 |
| c type lectin receptor signaling pathway | 104 | 0.5502737 | 1.8185351 | 0.00118126 |
| chemokine signaling pathway | 175 | 0.5309099 | 1.8029937 | 0.00137708 |
| tnf signaling pathway | 115 | 0.54467326 | 1.8014352 | 0.0013569 |
| tuberculosis | 157 | 0.5252922 | 1.8009368 | 0.00130036 |
| leukocyte transendothelial migration | 108 | 0.54386497 | 1.798611 | 0.00128547 |
| fc gamma r mediated phagocytosis | 93 | 0.5528731 | 1.798328 | 0.00123602 |
| il 17 signaling pathway | 80 | 0.55753464 | 1.7982724 | 0.00119025 |
| chagas disease | 99 | 0.5477854 | 1.787014 | 0.00131151 |
| viral protein interaction with cytokine and cytokine receptor | 80 | 0.5540851 | 1.7825427 | 0.00136086 |
| ribosome biogenesis in eukaryotes | 75 | 0.54645 | 1.7453148 | 0.00216595 |
| bacterial invasion of epithelial cells | 74 | 0.5318138 | 1.7097766 | 0.00354177 |
| b cell receptor signaling pathway | 77 | 0.5333845 | 1.6989267 | 0.00414681 |
| nod like receptor signaling pathway | 152 | 0.5038559 | 1.6942112 | 0.00441066 |
| acute myeloid leukemia | 65 | 0.53613573 | 1.6869932 | 0.00473883 |
| platelet activation | 120 | 0.50894314 | 1.6864316 | 0.00468223 |
| proteoglycans in cancer | 194 | 0.48938182 | 1.682394 | 0.00480546 |
| cytokine cytokine receptor interaction | 235 | 0.48301432 | 1.6693267 | 0.00588992 |
| human cytomegalovirus infection | 223 | 0.4790517 | 1.6623086 | 0.0064808 |
| sphingolipid signaling pathway | 121 | 0.49913174 | 1.6545153 | 0.00704192 |
| legionellosis | 55 | 0.53933156 | 1.6528103 | 0.00698066 |
| sphingolipid metabolism | 51 | 0.5456576 | 1.6480231 | 0.00728027 |
| salmonella infection | 235 | 0.47714943 | 1.6447738 | 0.00743476 |
| fc epsilon ri signaling pathway | 62 | 0.52954686 | 1.6435395 | 0.00738829 |
| cytosolic dna sensing pathway | 64 | 0.5211774 | 1.642739 | 0.0073449 |
| apoptosis | 125 | 0.49101418 | 1.6354749 | 0.00789384 |
| nucleocytoplasmic transport | 101 | 0.49485746 | 1.6343231 | 0.00780144 |
| protein export | 29 | 0.5775619 | 1.630342 | 0.00814337 |
| jak stat signaling pathway | 132 | 0.48256114 | 1.6234733 | 0.00875524 |
| inflammatory mediator regulation of trp channels | 97 | 0.48955125 | 1.6105975 | 0.01009291 |
| kaposi sarcoma associated herpesvirus infection | 189 | 0.46711066 | 1.595367 | 0.01223385 |
| viral life cycle hiv 1 | 57 | 0.51414174 | 1.594723 | 0.01213733 |
| biosynthesis of nucleotide sugars | 36 | 0.5515411 | 1.59105 | 0.01266123 |
| spliceosome | 129 | 0.47447425 | 1.5815196 | 0.01417101 |
| human immunodeficiency virus 1 infection | 205 | 0.46016434 | 1.575137 | 0.01520091 |
| necroptosis | 134 | 0.46633545 | 1.568847 | 0.01623965 |
| pancreatic cancer | 75 | 0.49070078 | 1.5624148 | 0.01735108 |
| influenza a | 149 | 0.4613068 | 1.5621598 | 0.01706259 |
| base excision repair | 44 | 0.51108277 | 1.547256 | 0.02093636 |
| phosphatidylinositol signaling system | 91 | 0.47685215 | 1.5413285 | 0.02221231 |
| hematopoietic cell lineage | 83 | 0.47740817 | 1.5340301 | 0.02410029 |
| viral carcinogenesis | 190 | 0.44836205 | 1.5329094 | 0.02405222 |
| hepatitis b | 146 | 0.45495212 | 1.5299218 | 0.02478306 |
| epstein barr virus infection | 203 | 0.4455037 | 1.529744 | 0.02446169 |
| egfr tyrosine kinase inhibitor resistance | 78 | 0.47532928 | 1.529581 | 0.02410809 |
| parathyroid hormone synthesis, secretion and action | 103 | 0.46193838 | 1.5289657 | 0.02386449 |
| regulation of actin cytoskeleton | 211 | 0.44777834 | 1.5276263 | 0.02390699 |
| amoebiasis | 86 | 0.47397637 | 1.5247401 | 0.02454909 |
| vegf signaling pathway | 58 | 0.48691338 | 1.518051 | 0.026202 |
| nucleotide excision repair | 56 | 0.48830962 | 1.5179724 | 0.02584889 |
| toxoplasmosis | 105 | 0.46229157 | 1.5172021 | 0.02580699 |
| tight junction | 160 | 0.44890684 | 1.5166571 | 0.0255722 |
| thyroid hormone synthesis | 67 | 0.48267278 | 1.516505 | 0.02528031 |
| dna replication | 34 | 0.52598906 | 1.5159863 | 0.02510999 |
| efferocytosis | 149 | 0.44599143 | 1.511619 | 0.02618464 |
| neutrophil extracellular trap formation | 142 | 0.4447911 | 1.5052389 | 0.02805513 |
| fanconi anemia pathway | 48 | 0.49874568 | 1.5049349 | 0.02774588 |
| neurotrophin signaling pathway | 116 | 0.4536626 | 1.4990883 | 0.0292274 |
| estrogen signaling pathway | 111 | 0.45171583 | 1.4975934 | 0.0293928 |
| hif 1 signaling pathway | 101 | 0.45374572 | 1.4964169 | 0.02944983 |
| alcoholic liver disease | 123 | 0.4463203 | 1.4957987 | 0.02934585 |
| ferroptosis | 40 | 0.4940308 | 1.479618 | 0.03537928 |
| p53 signaling pathway | 69 | 0.46765634 | 1.4762237 | 0.03633148 |
| chronic myeloid leukemia | 76 | 0.45939955 | 1.4729364 | 0.03727502 |
| proteasome | 46 | 0.48424035 | 1.4725589 | 0.03699487 |
| hepatitis c | 146 | 0.43758205 | 1.4710073 | 0.03722613 |
| fluid shear stress and atherosclerosis | 137 | 0.44044098 | 1.4700367 | 0.03716515 |
| inositol phosphate metabolism | 71 | 0.45836407 | 1.4667883 | 0.03832832 |
| thyroid cancer | 37 | 0.49950954 | 1.4562422 | 0.04260264 |
| pd l1 expression and pd 1 checkpoint pathway in cancer | 87 | 0.45075604 | 1.4551756 | 0.04270079 |
| non small cell lung cancer | 70 | 0.4571523 | 1.4530624 | 0.04331619 |
| cell cycle | 154 | 0.431812 | 1.4527456 | 0.04296188 |
| amino sugar and nucleotide sugar metabolism | 47 | 0.48140168 | 1.4518299 | 0.04291169 |
| aldosterone regulated sodium reabsorption | 36 | 0.5012284 | 1.4511614 | 0.0427565 |
| measles | 128 | 0.43332967 | 1.4511149 | 0.04233132 |
| glioma | 70 | 0.4537766 | 1.4505938 | 0.04209752 |
| oxytocin signaling pathway | 145 | 0.42385748 | 1.4426877 | 0.04557715 |
| phospholipase d signaling pathway | 141 | 0.43023664 | 1.4418789 | 0.04561702 |
| endocrine and other factor regulated calcium reabsorption | 52 | 0.46835744 | 1.4387329 | 0.04674064 |
| cholinergic synapse | 102 | 0.43292955 | 1.4307171 | 0.05096476 |
| bladder cancer | 37 | 0.48924762 | 1.4241095 | 0.05434711 |
| oocyte meiosis | 109 | 0.43246028 | 1.422497 | 0.05489382 |
| snare interactions in vesicular transport | 32 | 0.49633715 | 1.4168165 | 0.05774191 |
| transcriptional misregulation in cancer | 164 | 0.41955394 | 1.4156468 | 0.0579006 |
| gap junction | 78 | 0.43981594 | 1.4099351 | 0.06077263 |
| systemic lupus erythematosus | 84 | 0.43963698 | 1.4092172 | 0.06079619 |
| glycosphingolipid biosynthesis globo and isoglobo series | 16 | 0.5660833 | 1.4063346 | 0.06199051 |
| focal adhesion | 198 | 0.411648 | 1.4017122 | 0.06452136 |
| antigen processing and presentation | 74 | 0.4421508 | 1.3967718 | 0.0672138 |
| cytoskeleton in muscle cells | 213 | 0.40696198 | 1.3898485 | 0.07180528 |
| endocytosis | 256 | 0.40660295 | 1.3893187 | 0.07156873 |
| rna polymerase | 30 | 0.48875338 | 1.3853018 | 0.07406899 |
| growth hormone synthesis, secretion and action | 109 | 0.41843352 | 1.3848803 | 0.07374333 |
| small cell lung cancer | 87 | 0.42847073 | 1.3845967 | 0.07330206 |
| autophagy animal | 158 | 0.4110471 | 1.3839657 | 0.07310861 |
| glycosaminoglycan biosynthesis chondroitin sulfate / dermatan sulfate | 21 | 0.5267598 | 1.3787665 | 0.07668778 |
| synaptic vesicle cycle | 66 | 0.431768 | 1.3732692 | 0.08022554 |
| pi3k akt signaling pathway | 320 | 0.39164832 | 1.3708643 | 0.08139459 |
| rig i like receptor signaling pathway | 57 | 0.4443971 | 1.3681829 | 0.08272123 |
| melanoma | 65 | 0.4258314 | 1.3542794 | 0.09487889 |
| homologous recombination | 39 | 0.45950636 | 1.3520112 | 0.09609405 |
| tgf beta signaling pathway | 106 | 0.40881628 | 1.3475183 | 0.09964764 |
| cell adhesion molecules | 153 | 0.3991992 | 1.3467768 | 0.09961777 |
| mrna surveillance pathway | 89 | 0.41529444 | 1.345864 | 0.09967799 |
| age rage signaling pathway in diabetic complications | 100 | 0.41252625 | 1.3457525 | 0.09897011 |
| basal transcription factors | 39 | 0.45784608 | 1.3438543 | 0.10001165 |
| platinum drug resistance | 72 | 0.4200827 | 1.3342347 | 0.10894104 |
| terpenoid backbone biosynthesis | 23 | 0.498925 | 1.3309536 | 0.11154725 |
| glutathione metabolism | 58 | 0.4260305 | 1.3258799 | 0.11648479 |
| antifolate resistance | 29 | 0.4790708 | 1.3177818 | 0.1246732 |
| progesterone mediated oocyte maturation | 84 | 0.40409085 | 1.3125681 | 0.12953123 |
| serotonergic synapse | 100 | 0.39484876 | 1.3082035 | 0.13433848 |
| t cell receptor signaling pathway | 118 | 0.39081144 | 1.305657 | 0.13659771 |
| circadian entrainment | 89 | 0.40062547 | 1.3040206 | 0.1377463 |
| rap1 signaling pathway | 198 | 0.38198096 | 1.3026648 | 0.1384614 |
| apoptosis multiple species | 31 | 0.46835887 | 1.30261 | 0.13751045 |
| glycosaminoglycan biosynthesis heparan sulfate / heparin | 22 | 0.491283 | 1.3023673 | 0.136931 |
| axon guidance | 175 | 0.38188168 | 1.300317 | 0.13855796 |
| tyrosine metabolism | 28 | 0.45768538 | 1.2998558 | 0.13813679 |
| one carbon pool by folate | 19 | 0.50880635 | 1.2997699 | 0.13722895 |
| micrornas in cancer | 198 | 0.3799735 | 1.2996848 | 0.13634717 |
| mapk signaling pathway | 287 | 0.3742943 | 1.2958537 | 0.14055376 |
| human t cell leukemia virus 1 infection | 227 | 0.3758965 | 1.29569 | 0.13976437 |
| hepatocellular carcinoma | 160 | 0.38102144 | 1.2955102 | 0.13905062 |
| colorectal cancer | 85 | 0.40247104 | 1.2920029 | 0.14264105 |
| regulation of lipolysis in adipocytes | 50 | 0.42898855 | 1.2894721 | 0.14496554 |
| dilated cardiomyopathy | 96 | 0.39507654 | 1.287049 | 0.14696896 |
| glycosylphosphatidylinositol (gpi) anchor biosynthesis | 26 | 0.47190982 | 1.286691 | 0.14637418 |
| mineral absorption | 48 | 0.42880073 | 1.2864683 | 0.14570564 |
| ecm receptor interaction | 84 | 0.39815566 | 1.2820853 | 0.15052769 |
| endometrial cancer | 56 | 0.41258022 | 1.2786121 | 0.15404122 |
| renal cell carcinoma | 67 | 0.4017485 | 1.2758335 | 0.15698986 |
| adherens junction | 90 | 0.39099488 | 1.272435 | 0.16055734 |
| pathways in cancer | 482 | 0.36079773 | 1.27086 | 0.16187142 |
| cellular senescence | 167 | 0.3736482 | 1.2691804 | 0.16309799 |
| primary immunodeficiency | 31 | 0.44824824 | 1.2665089 | 0.16566478 |
| cholesterol metabolism | 44 | 0.4210898 | 1.2652878 | 0.16634633 |
| other types of o glycan biosynthesis | 41 | 0.42341223 | 1.2560407 | 0.17934103 |
| aldosterone synthesis and secretion | 89 | 0.38877806 | 1.2558483 | 0.17851312 |
| herpes simplex virus 1 infection | 302 | 0.35716322 | 1.2557715 | 0.17758064 |
| erbb signaling pathway | 84 | 0.38950318 | 1.2524168 | 0.18147916 |
| mitophagy animal | 93 | 0.38419434 | 1.2497754 | 0.18438117 |
| mtor signaling pathway | 147 | 0.36726263 | 1.2422774 | 0.1950998 |
| salivary secretion | 68 | 0.39217338 | 1.2385182 | 0.1996379 |
| arrhythmogenic right ventricular cardiomyopathy | 79 | 0.38544658 | 1.2376956 | 0.19974387 |
| gnrh signaling pathway | 84 | 0.3792664 | 1.2374319 | 0.19896592 |
| primary bile acid biosynthesis | 16 | 0.4925056 | 1.2336745 | 0.20389521 |
| mismatch repair | 22 | 0.47090027 | 1.2322987 | 0.20495011 |
| endocrine resistance | 90 | 0.37833855 | 1.2287569 | 0.2098248 |
| long term depression | 54 | 0.3980904 | 1.2269528 | 0.21166004 |
| human papillomavirus infection | 322 | 0.3517982 | 1.2261602 | 0.2118737 |
| th17 cell differentiation | 100 | 0.36988148 | 1.2245691 | 0.21317932 |
| glycerophospholipid metabolism | 95 | 0.36966065 | 1.2233511 | 0.21406044 |
| motor proteins | 169 | 0.36039707 | 1.2230151 | 0.21332565 |
| apelin signaling pathway | 131 | 0.36653724 | 1.2180063 | 0.22030291 |
| ras signaling pathway | 219 | 0.35266742 | 1.2157633 | 0.22270696 |
| inflammatory bowel disease | 54 | 0.39079386 | 1.2116544 | 0.22866412 |
| camp signaling pathway | 192 | 0.35122162 | 1.2108341 | 0.22882056 |
| glycerolipid metabolism | 58 | 0.390042 | 1.2103113 | 0.22843981 |
| choline metabolism in cancer | 95 | 0.3751967 | 1.2088976 | 0.22931764 |
| prolactin signaling pathway | 61 | 0.38066676 | 1.2081418 | 0.22932956 |
| insulin resistance | 104 | 0.36411312 | 1.2045056 | 0.23429306 |
| carbohydrate digestion and absorption | 37 | 0.41693154 | 1.2034068 | 0.23508346 |
| biosynthesis of amino acids | 74 | 0.37443972 | 1.2018446 | 0.23660766 |
| hypertrophic cardiomyopathy | 93 | 0.36914018 | 1.2008445 | 0.23699026 |
| intestinal immune network for iga production | 38 | 0.41323856 | 1.1984718 | 0.23978446 |
| ubiquitin mediated proteolysis | 139 | 0.35776109 | 1.1984093 | 0.23860863 |
| gastric acid secretion | 66 | 0.37902394 | 1.1937634 | 0.24598223 |
| ether lipid metabolism | 46 | 0.39432484 | 1.1926395 | 0.24665265 |
| arginine and proline metabolism | 49 | 0.3874266 | 1.1875814 | 0.25445598 |
| relaxin signaling pathway | 122 | 0.35515732 | 1.1850238 | 0.25777233 |
| insulin signaling pathway | 125 | 0.3498601 | 1.1845617 | 0.2573191 |
| collecting duct acid secretion | 25 | 0.44063744 | 1.1825109 | 0.25976488 |
| glutamatergic synapse | 103 | 0.35884038 | 1.1779492 | 0.26693615 |
| taurine and hypotaurine metabolism | 16 | 0.47636423 | 1.1762377 | 0.26887834 |
| bile secretion | 62 | 0.37577635 | 1.172859 | 0.27409917 |
| hippo signaling pathway | 145 | 0.34687558 | 1.1689688 | 0.28036922 |
| vitamin digestion and absorption | 22 | 0.4384999 | 1.163019 | 0.29105246 |
| gabaergic synapse | 83 | 0.36550477 | 1.1624508 | 0.2906322 |
| morphine addiction | 85 | 0.35876608 | 1.1580831 | 0.2982801 |
| vascular smooth muscle contraction | 128 | 0.34652835 | 1.1517866 | 0.30970725 |
| glycosphingolipid biosynthesis lacto and neolacto series | 25 | 0.41459402 | 1.1497489 | 0.3125604 |
| mucin type o glycan biosynthesis | 29 | 0.40372068 | 1.1440449 | 0.32320347 |
| dopaminergic synapse | 123 | 0.34249493 | 1.1389114 | 0.3323845 |
| nucleotide metabolism | 77 | 0.35429934 | 1.1357685 | 0.33746868 |
| foxo signaling pathway | 119 | 0.33980504 | 1.1334567 | 0.3407113 |
| viral myocarditis | 79 | 0.35471454 | 1.1324223 | 0.34137428 |
| chemical carcinogenesis receptor activation | 172 | 0.33290455 | 1.1321516 | 0.340278 |
| renin secretion | 65 | 0.36467403 | 1.1319115 | 0.33910096 |
| biosynthesis of cofactors | 134 | 0.33571798 | 1.125403 | 0.35176632 |
| steroid biosynthesis | 18 | 0.43241325 | 1.1182456 | 0.36578697 |
| adrenergic signaling in cardiomyocytes | 146 | 0.3312563 | 1.1179049 | 0.36479607 |
| central carbon metabolism in cancer | 65 | 0.35827297 | 1.116987 | 0.3650532 |
| type i diabetes mellitus | 54 | 0.36498448 | 1.1152539 | 0.3670786 |
| gastric cancer | 131 | 0.33420017 | 1.107887 | 0.3820539 |
| prostate cancer | 92 | 0.3400817 | 1.1023301 | 0.39313754 |
| insulin secretion | 77 | 0.34078172 | 1.096727 | 0.40396303 |
| longevity regulating pathway | 85 | 0.33782995 | 1.0948092 | 0.4064516 |
| asthma | 21 | 0.4166877 | 1.093724 | 0.40717372 |
| alcoholism | 137 | 0.32308748 | 1.0913559 | 0.41079137 |
| polycomb repressive complex | 77 | 0.3411706 | 1.0900993 | 0.41199273 |
| pyrimidine metabolism | 52 | 0.357594 | 1.0807244 | 0.4317135 |
| atp dependent chromatin remodeling | 109 | 0.326256 | 1.0750082 | 0.4431821 |
| drug metabolism other enzymes | 62 | 0.34010202 | 1.0596502 | 0.47855166 |
| vasopressin regulated water reabsorption | 41 | 0.35990724 | 1.0555822 | 0.4866509 |
| ovarian steroidogenesis | 44 | 0.3508899 | 1.0531682 | 0.49031466 |
| breast cancer | 133 | 0.31090048 | 1.0494946 | 0.4969537 |
| longevity regulating pathway multiple species | 57 | 0.3335558 | 1.0482726 | 0.49744642 |
| pancreatic secretion | 88 | 0.32339168 | 1.0388598 | 0.5183268 |
| folate biosynthesis | 25 | 0.38854438 | 1.0375232 | 0.5191704 |
| aminoacyl trna biosynthesis | 44 | 0.3522827 | 1.0369078 | 0.51836866 |
| th1 and th2 cell differentiation | 83 | 0.318795 | 1.0364147 | 0.5173218 |
| mannose type o glycan biosynthesis | 21 | 0.39926702 | 1.0356826 | 0.5168643 |
| cgmp pkg signaling pathway | 156 | 0.30267674 | 1.0235722 | 0.5442816 |
| allograft rejection | 47 | 0.33952695 | 1.0203263 | 0.5499065 |
| porphyrin metabolism | 29 | 0.36651063 | 1.0191395 | 0.5505447 |
| cysteine and methionine metabolism | 47 | 0.3425146 | 1.0163324 | 0.555044 |
| signaling pathways regulating pluripotency of stem cells | 123 | 0.29773068 | 0.9951721 | 0.6039046 |
| cortisol synthesis and secretion | 62 | 0.31653377 | 0.99297875 | 0.6066238 |
| circadian rhythm | 33 | 0.34255788 | 0.9889549 | 0.6136034 |
| cushing syndrome | 143 | 0.29133323 | 0.9876256 | 0.6141872 |
| melanogenesis | 87 | 0.3092013 | 0.987294 | 0.6123947 |
| galactose metabolism | 27 | 0.3539847 | 0.97876126 | 0.6300024 |
| autophagy other | 31 | 0.3421561 | 0.9743545 | 0.63784593 |
| graft versus host disease | 48 | 0.317367 | 0.96385676 | 0.65992385 |
| autoimmune thyroid disease | 49 | 0.31343275 | 0.9578847 | 0.6704253 |
| calcium signaling pathway | 220 | 0.27850297 | 0.9573039 | 0.66902137 |
| pentose and glucuronate interconversions | 19 | 0.36634475 | 0.9564837 | 0.66812307 |
| neuroactive ligand receptor interaction | 249 | 0.27229545 | 0.94044536 | 0.7013819 |
| other glycan degradation | 18 | 0.3648579 | 0.94014084 | 0.6991886 |
| nicotinate and nicotinamide metabolism | 38 | 0.32458326 | 0.94001406 | 0.6966414 |
| wnt signaling pathway | 158 | 0.2734094 | 0.9231463 | 0.72935677 |
| basal cell carcinoma | 55 | 0.30298638 | 0.9207584 | 0.7313948 |
| pantothenate and coa biosynthesis | 19 | 0.35944197 | 0.91805255 | 0.73387015 |
| gnrh secretion | 60 | 0.29400173 | 0.9159725 | 0.73505193 |
| amyotrophic lateral sclerosis | 341 | 0.25814614 | 0.9004498 | 0.7629808 |
| chemical carcinogenesis dna adducts | 41 | 0.29868844 | 0.8854463 | 0.7886314 |
| long term potentiation | 58 | 0.2864428 | 0.88531715 | 0.7858158 |
| histidine metabolism | 21 | 0.33343953 | 0.88335997 | 0.786299 |
| rna degradation | 77 | 0.2756038 | 0.8772583 | 0.7942584 |
| protein digestion and absorption | 90 | 0.26924753 | 0.8719924 | 0.8009308 |
| prion disease | 261 | 0.25148025 | 0.8682275 | 0.8042555 |
| hippo signaling pathway multiple species | 25 | 0.31557778 | 0.8555481 | 0.82269436 |
| adipocytokine signaling pathway | 69 | 0.268456 | 0.85285026 | 0.8242295 |
| fructose and mannose metabolism | 32 | 0.29767635 | 0.8415567 | 0.8390847 |
| thyroid hormone signaling pathway | 117 | 0.24919546 | 0.8218664 | 0.8651885 |
| hedgehog signaling pathway | 50 | 0.26743197 | 0.8179748 | 0.8678709 |
| lysine degradation | 59 | 0.25709617 | 0.8128427 | 0.8716486 |
| drug metabolism cytochrome p450 | 42 | 0.2716778 | 0.8019466 | 0.88297534 |
| amphetamine addiction | 62 | 0.2560162 | 0.79862607 | 0.88403124 |
| purine metabolism | 123 | 0.23151037 | 0.76967925 | 0.91603434 |
| alzheimer disease | 361 | 0.21829155 | 0.7690669 | 0.9133709 |
| type ii diabetes mellitus | 43 | 0.25699764 | 0.7670233 | 0.9122464 |
| steroid hormone biosynthesis | 39 | 0.26160973 | 0.76435524 | 0.91170007 |
| notch signaling pathway | 59 | 0.243087 | 0.76034546 | 0.9125359 |
| phototransduction | 20 | 0.29178724 | 0.75368094 | 0.91596097 |
| ampk signaling pathway | 120 | 0.22363053 | 0.7503256 | 0.91627115 |
| pathways of neurodegeneration multiple diseases | 444 | 0.21262522 | 0.746714 | 0.9162633 |
| selenocompound metabolism | 17 | 0.29119056 | 0.7273551 | 0.9305689 |
| parkinson disease | 253 | 0.20852207 | 0.72123706 | 0.9323193 |
| abc transporters | 46 | 0.2411038 | 0.7168711 | 0.93252057 |
| glucagon signaling pathway | 95 | 0.20594755 | 0.672353 | 0.9595168 |
| cocaine addiction | 42 | 0.21669604 | 0.64587754 | 0.97010297 |
| renin angiotensin system | 26 | 0.22789013 | 0.6235992 | 0.9759485 |
